# Developmental differences in canonical cortical networks: Insights from microstructure-informed tractography

**DOI:** 10.1162/netn_a_00378

**Published:** 2024-10-01

**Authors:** Sila Genc, Simona Schiavi, Maxime Chamberland, Chantal M. W. Tax, Erika P. Raven, Alessandro Daducci, Derek K. Jones

**Affiliations:** Cardiff University Brain Research Imaging Centre (CUBRIC), School of Psychology, Cardiff University, Cardiff, United Kingdom; Neuroscience Advanced Clinical Imaging Service (NACIS), Department of Neurosurgery, The Royal Children’s Hospital, Parkville, Victoria, Australia; Developmental Imaging, Clinical Sciences, Murdoch Children’s Research Institute, Parkville, Victoria, Australia; Department of Computer Science, University of Verona, Italy; ASG Superconductors, Genova, Italy; Eindhoven University of Technology, Department of Mathematics and Computer Science, Eindhoven, Netherlands; Image Sciences Institute, University Medical Center Utrecht, Utrecht, Netherlands; Cardiff University Brain Research Imaging Centre (CUBRIC), School of Physics and Astronomy, Cardiff University, United Kingdom; Center for Biomedical Imaging, Department of Radiology, New York University Grossman School of Medicine, New York, NY, USA

**Keywords:** Development, Connectivity, Microstructure-informed tractography, Cortical, Diffusion

## Abstract

In response to a growing interest in refining brain connectivity assessments, this study focuses on integrating white matter fiber-specific microstructural properties into structural connectomes. Spanning ages 8–19 years in a developmental sample, it explores age-related patterns of microstructure-informed network properties at both local and global scales. First, the diffusion-weighted signal fraction associated with each tractography-reconstructed streamline was constructed. Subsequently, the convex optimization modeling for microstructure-informed tractography (COMMIT) approach was employed to generate microstructure-informed connectomes from diffusion MRI data. To complete the investigation, network characteristics within eight functionally defined networks (visual, somatomotor, dorsal attention, ventral attention, limbic, fronto-parietal, default mode, and subcortical networks) were evaluated. The findings underscore a consistent increase in global efficiency across child and adolescent development within the visual, somatomotor, and default mode networks (*p* < 0.005). Additionally, mean strength exhibits an upward trend in the somatomotor and visual networks (*p* < 0.001). Notably, nodes within the dorsal and ventral visual pathways manifest substantial age-dependent changes in local efficiency, aligning with existing evidence of extended maturation in these pathways. The outcomes strongly support the notion of a prolonged developmental trajectory for visual association cortices. This study contributes valuable insights into the nuanced dynamics of microstructure-informed brain connectivity throughout different developmental stages.

## Author Summary

There is a growing interest in incorporating biologically relevant white matter properties into the analysis of brain networks to obtain a more quantitative assessment of brain connectivity. In a developmental sample aged 8–19 years, we studied age-related patterns of local and global network properties. We generated microstructure-informed connectomes using diffusion MRI data, and computed network characteristics in eight functionally defined networks (visual, somatomotor, dorsal attention, ventral attention, limbic, fronto-parietal, default mode, and subcortical networks). The findings reveal that throughout child and adolescent development, global efficiency increases in the visual, somatomotor, and default mode networks, and mean strength increases in the somatomotor and visual networks. Nodes belonging to the dorsal and ventral visual pathways demonstrate the largest age-dependence in local efficiency, supporting previous evidence of protracted maturation of dorsal and ventral visual pathways. The results provide compelling evidence that there is a prolonged development of visual association cortices.

## INTRODUCTION

The transition from childhood to adolescence is a period of profound neurobiological and cognitive development where the human brain undergoes significant changes to refine neural substrates prior to adulthood (Blakemore & Choudhury, 2006). Essential to this process are the white matter pathways that form a structural scaffold facilitating connections and communication between cortical regions. Their development follows a stereotypical pattern of myelination, which closely mirrors the functional capacity of neural systems. For example, primary sensory, motor, and visual pathways typically complete myelination by the first two years of life (Deoni et al., 2015), whereas frontal and temporal association regions continue to develop well into adulthood, with peak myelination happening in the second decade of life (Bartzokis et al., 2012; Yakovlev & Lecours, 1967). The process of axonal development is less clear, with early *ex vivo* studies indicating stabilization of corpus callosum axonal count by six months of age (LaMantia & Rakic, 1990) and further work indicating changes to axonal and myelin properties at pubertal onset (Genc et al., 2023; Juraska & Willing, 2017; Paus, 2010).

Developmental studies using magnetic resonance imaging (MRI) have revealed that white matter volume steadily increases over childhood and adolescence (Giedd et al., 1999; Lenroot & Giedd, 2006), likely by way of coupled radial growth of the axon and myelin sheath. In tandem, functional MRI (fMRI) studies suggest a greater degree of temporal network connectivity, which remodels from infancy to early adulthood (Grayson & Fair, 2017). Early in childhood, sensorimotor systems become well integrated and coordinated, and show little change into adulthood (Gu et al., 2015). Later in adolescence, functional hubs such as fronto-parietal, attentional, and salience networks become increasingly segregated, allowing for flexibility as the adolescent brain becomes more adaptable to increase performance and efficiency (Bassett, Wymbs, et al., 2011).

Diffusion magnetic resonance imaging (dMRI) has enabled novel discoveries in spatial and temporal patterns of white matter fiber development (Geeraert et al., 2019; Genc et al., 2018; Herting et al., 2017; Lebel & Beaulieu, 2011; Palmer et al., 2022; Tamnes et al., 2018). Structural connectivity has been studied using diffusion MRI tractography (Hagmann et al., 2007) to reconstruct white matter pathways or connections between nodes of interest (e.g., between distinct predefined cortical regions). Connection strength is commonly defined using streamline count, that is, the number of streamlines, derived from tractography, that run between nodes. However, this notion can be arbitrary, since streamline count is not biologically informative and can heavily depend on acquisition and processing parameters (Jones et al., 2013; Yeh et al., 2021; Zhang et al., 2022). Recent studies have attempted to improve the status quo in determining biologically informative determinants of connection strength using diffusion MRI (R. Smith et al., 2022; Zhang et al., 2022). However, the question remains, which measures are optimally informative?

To define more informative edge weights for the structural connectome, the “tractometry” approach was introduced in Bells et al. (2011), Jones et al. (2006), and Kanaan et al. (2006) and employed to study typical white matter development (Chamberland et al., 2019). This approach includes the mapping of microstructural measures along tractography-reconstructed pathways and computing average values for quantitative comparisons between measures. A challenge arises when multiple bundles pass through the same imaging voxel (an extremely prevalent phenomenon; see Jeurissen et al., 2013; Schilling et al., 2022), leading to biased measures assigned to each constituent bundle (Schiavi et al., 2022). The convex optimization modeling for microstructure-informed tractography (COMMIT; Daducci et al., 2013, 2015) approach addresses this problem by deconvolving specific microstructural features on each streamline to recover individual contributions to the measured signal. By replacing the commonly used streamline count with intra-axonal signal fraction (IASF), it offers a quantitative and more biologically informative assessment of brain connectivity (Bergamino et al., 2022; Gabusi et al., 2022; Schiavi et al., 2022; Schiavi, Ocampo-Pineda, et al., 2020; Schiavi, Petracca, et al., 2020).

To investigate age-related differences in structural connectivity among various canonical or domain-specific networks, graph theory provides a powerful analytical tool (Fornito et al., 2016; Zhang et al., 2022). Graph theoretical analysis permits the computation of networks at different levels of organization (Fornito et al., 2016; Yeh et al., 2021), using measures classified as (a) local, quantifying properties of individual nodes; (b) mesoscale, describing interconnected clusters of nodes; and (c) global, describing whole-brain connectivity properties (Fornito et al., 2016; Rubinov & Sporns, 2010). At the global scale, graph measures reveal how the brain’s structural wiring facilitates information communication between distant regions and cognitive systems. While structurally connected regions can communicate directly, signal propagation between unconnected nodes requires a sequence of one or more intermediate connections (Zhang et al., 2022). Thus, investigating these measures across and between predefined cognitive systems during development can shed light on the structural mechanisms behind functional expression (Seguin et al., 2019).

Given that it has been shown that white matter microstructure, at the voxel and tract level, continues to develop well into the third decade of life (Lebel & Beaulieu, 2011; Lebel & Deoni, 2018), we were interested in studying how *network* properties mature from childhood to adolescence when weighted by their microstructural properties. Here we construct microstructure-informed connectomes and study age-related patterns of commonly used local and global structural brain network properties in a typically developing sample aged 8–19 years.

## MATERIALS AND METHODS

### Participants

We enrolled a sample of typically developing children and adolescents aged 8–19 years recruited as part of the Cardiff University Brain Research Imaging Centre (CUBRIC) Kids study, with ethical approval from the School of Psychology ethics committee at Cardiff University. Participants and their parents/guardians were recruited via public outreach events, and written informed consent was obtained from the primary caregiver of each child participating in the study. Adolescents aged 16–19 years additionally provided written consent. Children were excluded from the study if they had nonremovable metal implants, or a reported history of a major head injury or epilepsy. All procedures were conducted in accordance with the Declaration of Helsinki. A total of 88 children (mean age = 12.6, *SD* = 2.9 years) were included in the current study (46 female).

### MRI Acquisition

Images were acquired on a 3T Siemens Connectom system with ultra-strong (300 mT/m) gradients. As described in Genc et al. (2020), the protocol comprised (a) a 3D magnetization prepared rapid gradient echo (MPRAGE) for structural segmentation (TE/TR = 2/2,300 ms; voxel size 1 × 1 × 1 mm^3^); (b) multi-shell dMRI acquisition (TE/TR = 59/3,000 ms; voxel size = 2 × 2 × 2 mm^3^) with b∈[500, 1,200, 2,400, 4,000, 6,000] s/mm^2^ in 30, 30, 60, 60, 60 directions, respectively, and additional 14 b = 0 s/mm^2^ volumes. Diffusion MRI data were acquired in an anterior-posterior phase-encoding direction, with one additional posterior-anterior volume.

### MRI Processing

**Figure 1.**
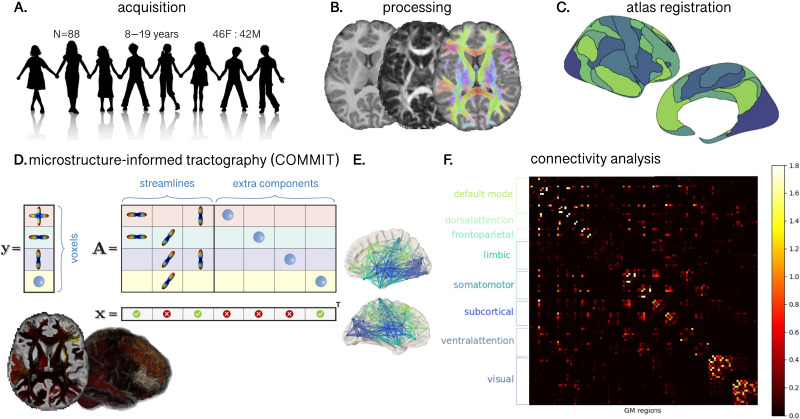
Workflow for constructing structural connectivity networks based on COMMIT-derived streamline weights. (A) MRI data were acquired on a 3T system with 300 mT/m gradients. (B) T1 and dMRI data were preprocessed. (C) Canonical cortical networks derived from a functional atlas (Yeo et al., 2011) were coregistered to individual subject space. (D) COMMIT (Daducci et al., 2013, 2015) was applied using a stick-zeppelin-ball model to filter out implausible connections, where computed weights reflect the intra-axonal signal fraction of each connection (brighter values = higher IASF). (E) Interconnected nodes colored by canonical cortical network. (F) Connectivity matrix demonstrating connection strength between nodes within each network (brighter values = higher IASF).

**Figure 2.**
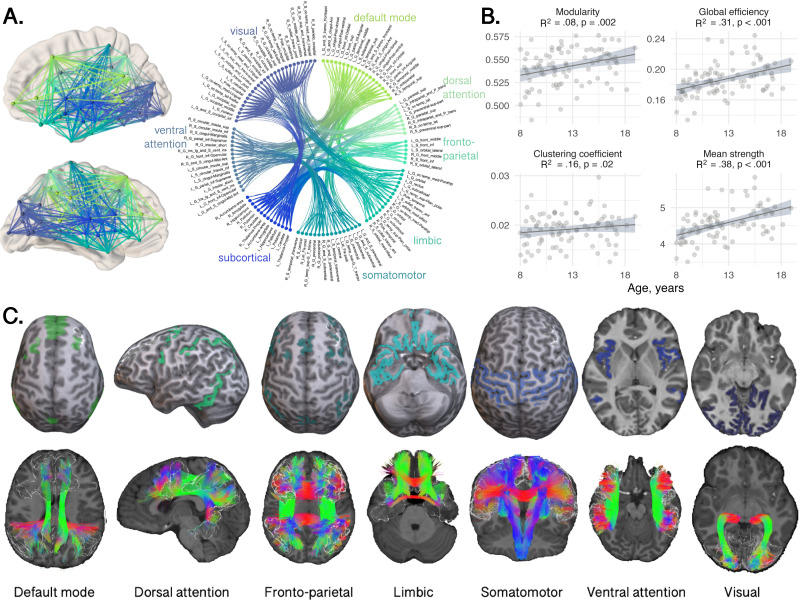
Relationship between age and global network measures computed for the whole connectome realized with Destrieux parcellation. (A) Interconnected nodes obtained using the intra-axonal signal fraction estimated with COMMIT, colored by canonical cortical network. (B) Association between age and network characteristics between networks (*R*^2^ and *p* value). (C) Depiction of atlas-derived cortical functional networks and representative white matter tracts traversing these networks, for an 8-year-old female participant.

**Figure 3.**
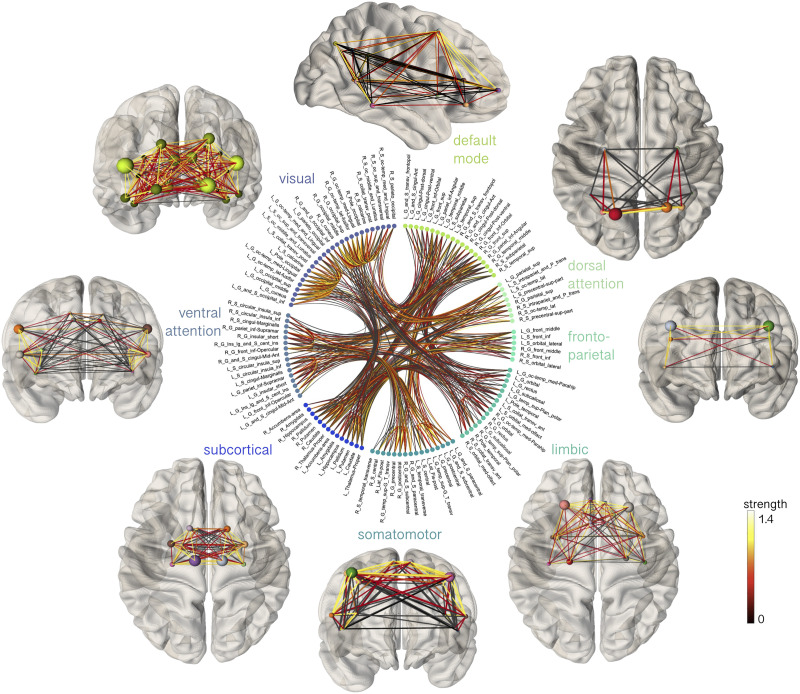
Spatial representation of the eight canonical cortical networks, with connections between nodes colored by strength.

A summary of image processing steps is illustrated in Figure 1. T1-weighted data were processed using FreeSurfer version 6.0 (https://surfer.nmr.mgh.harvard.edu) to derive a white matter mask and parcellate the cortical gray matter according to the Destrieux atlas (Destrieux et al., 2010). Next, we registered the Yeo functional atlas (Yeo et al., 2011) in MNI space to each individual subject’s space using a nonlinear transformation as implemented in FNIRT of FSL (S. M. Smith et al., 2004). This procedure allowed us to obtain eight functionally relevant cortical canonical networks (herein referred to as “Yeo7”) for further interrogation (visual, somatomotor, dorsal attention, ventral attention, limbic, fronto-parietal, default mode network, subcortical). Subsequently, we grouped regions of interest (ROIs) from the Destrieux atlas into the eight Yeo atlas networks. To merge the two atlases within each subject, we employed a data-driven approach (see Baum et al., 2017). Briefly, each parcellated brain region was assigned to one of eight canonical functional brain networks (Yeo et al., 2011) by considering the maximum number of voxels in the intersection between the masks. We ensured that the same overlap was confirmed in the homologous ROIs and for at least 80% of the enrolled subjects, discarding any Destrieux ROIs that did not meet these criteria. The final subdivision can be seen in Figures 2 and 3 and Supporting Information Table S2. Finally, we linearly registered the T1-weighted images and the corresponding parcellations on dMRI data using FLIRT (Jenkinson et al., 2002) with boundary-based optimization (Greve & Fischl, 2009). To investigate whether any result was robust against atlas choice, we repeated the same process with cortical parcellation using the Desikan-Killiany atlas (Desikan et al., 2006) and by grouping nodes into five distinct lobes (frontal, parietal, temporal, occipital, subcortical).

Diffusion MRI data were preprocessed as detailed in Genc et al. (2020). Briefly, the preprocessing pipeline involved FSL (S. M. Smith et al., 2004), MRtrix3 (Tournier et al., 2019), and ANTs (Avants et al., 2011) tools using the following steps: denoising (Veraart et al., 2016); slice-wise outlier detection (Sairanen et al., 2018); correction for drift (Vos et al., 2017); motion, eddy, and susceptibility-induced distortions (Andersson et al., 2003; Andersson & Sotiropoulos, 2016); Gibbs ringing artifact (Kellner et al., 2016); bias field (Tustison et al., 2010); and gradient non-uniformities (Glasser et al., 2013; Rudrapatna et al., 2021). We performed multi-shell, multi-tissue constrained spherical deconvolution (MSMT-CSD; Jeurissen et al., 2014) and generated a whole-brain probabilistic tractogram seeding from the white matter comprising 3 million streamlines (Tournier et al., 2010).

We then applied COMMIT (Daducci et al., 2013, 2015) using a stick-zeppelin-ball model (Panagiotaki et al., 2012) to effectively filter out implausible connections while obtaining the intra-axonal signal fraction for each streamline, as described in Schiavi, Petracca, et al. (2020). For a set of fixed intra- and extra-axonal diffusivities, we assume that the IASF is constant along the streamline. To set the diffusivity parameters in COMMIT, we performed voxel-wise estimations in one younger participant (8-year-old female) and one older participant (17-year-old female). In the white matter, diffusivities had minimal variation between the younger and older participant (Supporting Information Table S1). As a result, for all subjects we set the following diffusivities d_par_ = d_par_zep_ = 1.7 × 10^−3^ mm^2^/s, d_perp_ = 0.61 × 10^−3^ mm^2^/s, d_iso_ in [1.7, 3.0] × 10^−3^ mm^2^/s for all participants.

For each subject, the connectomes were built using nodes from the individual T1-based parcellation by assigning the total IASF associated to each bundle as edge weights as in Schiavi, Petracca, et al. (2020) and Gabusi et al. (2022). Briefly, for each subject, the microstructure-informed connectomes (i.e., obtained using COMMIT weights reflecting IASF associated to each streamline as entries) were built using the gray matter parcellation described above and computing the weighted average intra-axonal signal contribution of each bundle:

$$a_{ij}=\frac{\Sigma_{k=1}^{N_{ij}}x_{ij}^{k}\cdot l_{k}}{\frac{\Sigma_{k=1}^{N_{ij}}l_{k}}{N_{ij}}},$$

where *i*, *j* are the indices of ROIs connected by the bundle; *N*_*ij*_ is bundle’s number of streamlines; $x_{ij}^{k}$ is the weight of the streamline, *k*, obtained by COMMIT, and *l*_*k*_, its length. In this way, each entry contained the total IASF associated to the bundle given by the weighted average of the streamline contribution multiplied by its length and divided by the average length of the bundle.

### Network Analysis

To investigate the relationship between network characteristics and age, we used the Brain Connectivity Toolbox for Python (Rubinov & Sporns, 2010) to compute the following weighted network measures:

- modularity according to Newman’s spectral community detection (Newman, 2013) with resolution parameter gamma = 1;
- global efficiency as the average of the inverse shortest path length (Rubinov & Sporns, 2010);
- local efficiency as the global efficiency computed on the neighborhood of the node (Rubinov & Sporns, 2010);
- clustering coefficient as the mean of a node’s clustering coefficient computed as the average intensity of triangles around each node; and
- mean strength as the average of all the nodal strengths, computed as the sum of the weights of links connected to the node.

We computed these global network measures for the entire connectome, and again using smaller graphs containing only the nodes within each subnetwork of the Yeo7 atlas.

### Age Relationships

To investigate age-related patterns of network characteristics across the eight Yeo7 networks and five lobes, we applied linear mixed-effects modeling using lme4 (Bates et al., 2015) in R (RStudio v3.4.3). We built a linear model that included age (linear term), sex, and Yeo7 network as predictors, with intracranial volume (ICV) included as a covariate. We examined four network characteristics (modularity, global efficiency, clustering coefficient, mean strength) and compared the fit of the standard linear model with alternative models that incorporated interaction terms. To identify the most appropriate model, we used the Akaike information criterion (AIC; Akaike, 1974), selecting the model with the lowest AIC as the most parsimonious. Individual general linear models were run to determine age-related differences in specific network characteristics in all eight Yeo7 networks. Evidence for an association was deemed statistically significant when *p* < 0.005 (Benjamin et al., 2018).

### Feature Importance

To identify locally important nodes that contribute to developmental patterns within networks (identified in the previous section), we performed age prediction using linear regression and ElasticNet regularization in scikit-learn (i.e., L1 and L2 penalties). We investigated feature importance using the ROIs comprised in each network for age prediction of local efficiency. First, we randomly split the data into training and validation sets using an 80:20 ratio, resulting in 80% of the data being allocated for training purposes and the remaining 20% for model evaluation (total *N* = 88: 70 training; 18 testing). Then, we performed feature scaling to ensure that all variables were on a similar scale. To assess the generalization performance of the ElasticNet model and to prevent overfitting, we employed a fivefold cross-validation approach. We performed a grid search to determine the optimal values for the L1 ratio ([0.1, 0.5, 0.7, 0.9, 0.95, 0.99, 1]) based on the regression coefficient (*R*^2^).

The performance of the model was assessed using the validation dataset. Finally, the features with the largest weight coefficients were extracted to identify specific cortical regions driving age relationships in local network efficiency.

## RESULTS

### Global Network Characteristics

Linear models revealed a positive relationship between age and modularity (*R*^2^ = 0.08, *p* = 0.002), global efficiency (*R*^2^ = 0.31, *p* < 0.001), and mean strength (*R*^2^ = 0.38, *p* < 0.001) (Figure 2B). The relationship between age and clustering coefficient was not statistically significant (*R*^2^ = 0.16, *p* = 0.02). As shown in the circle plot in Figure 2A, we also noted strong intraregional connectivity and strength within the visual and somatomotor networks, indicating robust interactions among regions within these networks.

To test whether specific networks were driving these developmental patterns of network properties, we tested age-by-network interactions using a linear mixed-effects model. The various models tested and the model selection results are summarized in Supporting Information Table S3. The best fitting model for all four graph measures included an age-by-network-by-sex interaction term. We observed significant age-by-network interactions in modularity (*F* = 6.6, *p* < 0.001), global efficiency (*F* = 6.7, *p* < 0.001), clustering coefficient (*F* = 3.3, *p* = 0.002), and mean strength (*F* = 23.9, *p* < 0.001). As these results indicated that there were age-related differences in network properties *between* the networks, we performed subsequent analyses to test for age associations *within* networks, to discern whether developmental patterns differed regionally. The various networks tested and their corresponding anatomical tractography depictions are illustrated in Figure 2C.

### Subnetwork Characteristics

**Figure 4.**
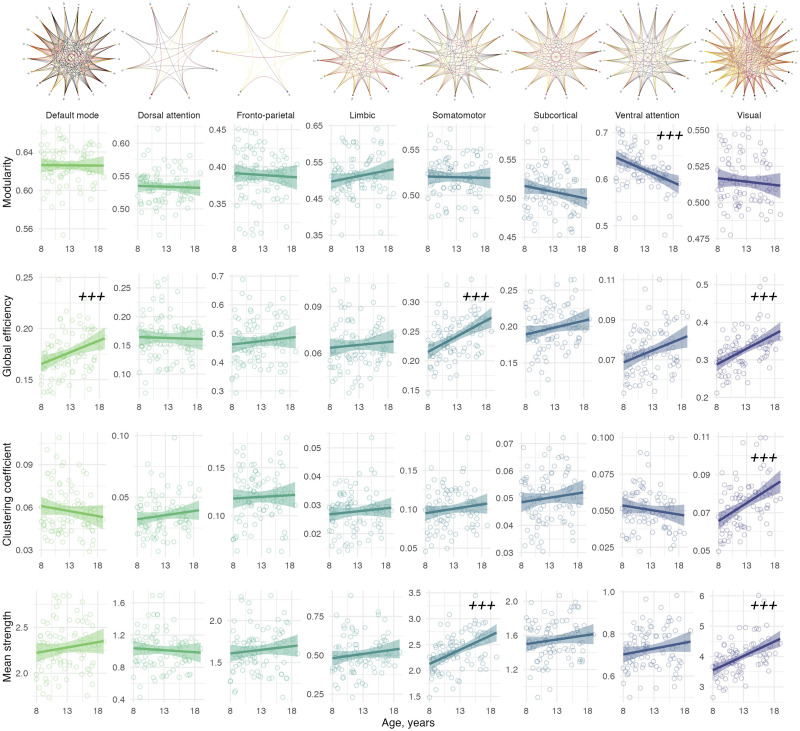
Association between age and network properties within subnetworks. Significant age relationships are annotated (+++ denotes *p* < 0.005). Top panel represents circle plots of within-network nodes, with brighter yellow connections indicative of higher mean strength. Nodes within the circle plots are labeled by number (see Supporting Information Table S2).

We identified regional differences in the age-related development of specific subnetworks (Table 1 and Figure 4). Through linear regression analyses within individual networks, we found statistically significant relationships between age and global efficiency in the default mode (*R*^2^ = 0.38, *p* = 0.001), somatomotor (*R*^2^ = 0.28, *p* < 0.001), and visual networks (*R*^2^ = 0.43, *p* < 0.001). Clustering coefficient was positively associated with age in the visual network (*R*^2^ = 0.37, *p* < 0.001). Moreover, age exhibited a positive association with mean strength in the somatomotor network (*R*^2^ = 0.33, *p* < 0.001) and the visual network (*R*^2^ = 0.46, *p* < 0.001). We also observed a negative association between age and modularity in the ventral attention network (*R*^2^ = 0.13, *p* < 0.001). These results were replicated when including connection density as a covariate to each linear model, with the additional correlation observed between clustering coefficient and age in the somatomotor network (*R*^2^ = 0.63, *p* < 0.001; Supporting Information Table S4). Overall, our results highlight the distinct age-related developmental patterns in the visual and somatomotor networks.

**Table 1.** Summary statistics for the relationship between age and global subnetwork characteristics. Adjusted *R*^2^ determined using a linear model including age, sex, and total intracranial volume. **Bold** values indicate *p* < 0.005. † denotes a significant difference in the slope of the age relationship compared with the visual network.

Network	Modularity		Global efficiency		Clustering coefficient		Mean strength	
	*R* ^2^	*p* value	*R* ^2^	*p* value	*R* ^2^	*p* value	*R* ^2^	*p* value
Default mode	0.04	0.55	0.38	**0.001**	0.10	0.59†	0.43	0.13†
Dorsal attention	−0.03	0.81	0.06	0.41†	0.09	0.20	0.06	0.23†
Fronto-parietal	0.07	0.66	0.03	0.58	−0.01	0.96	0.07	0.51†
Limbic	0.07	0.14	0.19	0.92	0.14	0.81	0.21	0.53†
Somatomotor	0.01	0.75	0.28	**<0.001**	0.30	0.20	0.33	**<0.001**
Subcortical	0.08	0.27	0.03	0.26	0.01	0.72	0.02	0.47†
Ventral attention	0.13	**<0.001**	0.19	0.006	0.11	0.47†	0.22	0.12†
Visual	0.11	0.17	0.43	**<0.001**	0.37	**<0.001**	0.46	**<0.001**

Sex differences were observed, where males had higher clustering coefficient in the visual network (*β*[95%CI] = 0.67 [0.29, 1.06], *p* = 0.0009), and higher mean strength in the default mode network (*β*[95%CI] = 0.71 [0.34, 1.08], *p* = 0.0002), compared with females. Sex interactions (slope of M > F) were apparent in modularity of the limbic network (*β*[95%CI] = 0.74 [0.31, 1.17], *p* = 0.0009).

To confirm that the age dependence of visual network properties were significantly different from other networks, we performed linear mixed-effects modeling to discern whether age-by-network interactions were significantly different between the visual network and the seven remaining subnetworks. Where the age relationship in the visual network was significantly stronger than each subsequent network, this is summarized in Supporting Information Table S5 and annotated in Table 1. In summary, the most marked observations were in network strength, where the visual network had a significantly stronger age dependency compared with each individual network, apart from the somatomotor network, which also had a positive relationship with age.

### Feature Importance of Local Efficiency

**Figure 5.**
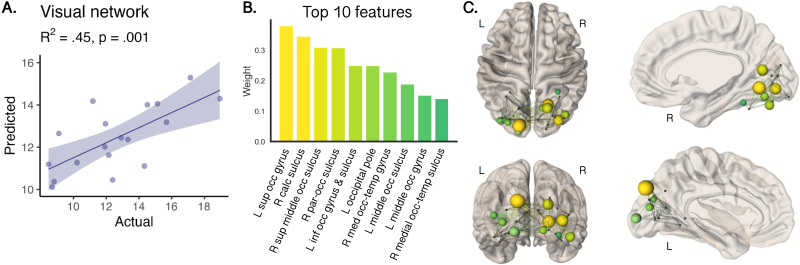
Feature importance for age prediction of local network efficiency in the visual cortex. (A) Predicted age was significantly associated with actual age. (B) Top 10 regions that contributed most to age-related patterns displayed in panel C. (C) Axial, sagittal, and coronal glass brain views, where nodes are scaled and color-coded by weight. Nodes with high feature importance included left superior and middle occipital gyrus and right medial occipito-temporal gyrus.

Age prediction of local efficiency in the visual network yielded a regression coefficient of 0.45 (root mean square error: 2.2, *p* = 0.001, Figure 5A) on the validation set (optimal value for L1 = 0.1). Feature importance in the visual network identified specific nodes (Figure 5) driving age-related increases in local efficiency. The 10 most sensitive nodes were balanced between hemispheres (5 nodes in right hemisphere, and 5 in the left) and accounted for 75% of variation in total weights (of a total of 26 nodes). Figure 5B summarizes the regions ranked by weight, and Figure 5C depicts these regions in axial, sagittal, and coronal views in 3D. Nodes with high feature importance for age clustered together, including nodes that form the dorsal (left superior occipital gyrus and middle occipital gyrus and sulcus) and the ventral (right medial occipito-temporal sulcus and gyrus, and right lingual gyrus) visual pathways.

Age prediction for local efficiency of the somatomotor network yielded a weaker regression coefficient of 0.10 that was not statistically significant (*p* = 0.10). Feature importance identified specific regions driving age-related increases in local efficiency. Six nodes balanced between hemispheres (three nodes in right hemisphere, and three in the left) accounted for 70% of the variation in total weights (of a total of 16 nodes). Nodes with high feature importance for age included the bilateral precentral gyrus, right postcentral gyrus, bilateral central sulcus, and left transverse temporal gyrus.

## DISCUSSION

We used microstructure-informed tractography to investigate global and local network characteristics in canonical cortical networks among a group of typically developing children and adolescents. Our study revealed three main findings:

First, whole-brain network-based measures of modularity, global efficiency, and mean strength increased with age. This indicates that as children move through adolescence, the shortest path between nodes (in this case, regions from the Destrieux parcellation) decreases, resulting in a more efficient transfer of information. As a result, the nodes tend to cluster together to form hubs, and the strength of each white matter connection increases with age. These findings align with known age-related increases in global efficiency during adolescent development (Baker et al., 2015; Khundrakpam et al., 2013; Koenis et al., 2018; van den Heuvel & Sporns, 2013). Additionally, previous white matter studies have shown substantial increases in intra-axonal signal fraction with age (Chang et al., 2015; Genc et al., 2020; Palmer et al., 2022), aligning with our observations of age-related increases in mean strength.

Second, subnetwork analyses revealed specific networks with substantial age-related differences occurring from childhood to adolescence. In the default mode, somatomotor, and visual networks, global efficiency was higher with older age. Additionally, clustering coefficient was higher with age in the visual network, and mean strength was higher with age in the somatomotor and visual networks. Notably, brain structures such as the primary visual and somatomotor cortex have highly organized and specialized structures that are closely related to their function, such as discriminating visual features (Wandell, 1999) and performing specific motor functions (Gordon et al., 2023).

Together, our findings of age-related maturation of network efficiency and strength suggests a high degree of integration and communication within motor and visual processing regions, potentially reflecting the ongoing maturation of visual information processing and motor coordination capabilities during development. Our specific findings in the visual network align with previously observed temporal patterns of white matter microstructural maturation in the visual cortex (Colby et al., 2011; Genc et al., 2017) that are likely to be closely linked to age-related increases in axon density in humans (Genc et al., 2020) and rodents (Juraska & Willing, 2017).

Age prediction in the visual cortex pointed to a smaller cluster of five regions per hemisphere that contributed to more than 75% of the observed age-related differences in local network efficiency. Our data-driven approach suggests that connections between nodes in the left dorsal (middle and superior occipital) visual pathway and the right ventral (middle occipito-temporal) visual pathway are driving developmental improvements in local network efficiency. The visual system undergoes early establishment during prenatal development and continues to mature through life (Gogtay et al., 2004; Knudsen, 2004). While myelination in the visual cortex is largely completed by the first year of life (Deoni et al., 2015), recent research indicates that myelination follows a protracted course in ventral temporal cortices (Natu et al., 2019). Ongoing intra-cortical myelination of the ventral temporal cortex may underlie MRI-derived estimates of cortical thinning, previously attributed to synaptic pruning (Gomez et al., 2017; Natu et al., 2019).

The maturation of association visual cortices supports higher level visual processing (e.g., recognizing and discriminating objects, motion perception; Gomez et al., 2018). Our findings align with task-based fMRI studies involving object and shape recognition tasks, which demonstrate protracted development of dorsal and ventral visual pathways (Freud et al., 2019; Ward et al., 2023). These developmental improvements in shape-processing mechanisms likely contribute to microstructure-specific strengthening of global network efficiency and strength of white matter connections within the visual network through child and adolescent brain development. The age-related increases in local network efficiency in lateral temporo-occipital cortices may facilitate improvements in visual processing and function between these association cortices.

The myelination of these visual pathways may help to refine and optimize the neural connections and improve visual processing capabilities. While we did not directly study myelination here, the intra-axonal signal fraction explains a significant proportion of the age-related variance in network efficiency and connection strength. Taken together, our findings suggest that white matter connections within the visual cortex undergoes protracted development through childhood and adolescence. While our study primarily focuses on white matter microstructure for exploring graph-based measures, our observations of higher efficiency and connection strength with older age is predominantly due to ongoing microstructural maturation in the visual cortex.

### Methodological Advantages of the Current Approach

We employed a data-driven approach to establish correspondence between a structural parcellation and functional atlas in each participant (Baum et al., 2017). This approach involved selecting the maximum number of voxels in the intersection between a smaller cortical region with its corresponding larger functional network. By ensuring that this overlap was consistent with the homologous ROIs and in at least 80% of the participants, we generated canonical cortical networks for the basis of regional graph-based analyses.

One of the significant advantages of the COMMIT framework is its ability to assign specific microstructural properties to individual tractography-reconstructed streamlines, which sets it apart from conventional (voxel-wise or vertex-wise) approaches. Without taking these factors into account, complex intra-voxel heterogeneity (Schilling et al., 2022) and nodal size (Bassett, Brown, et al., 2011) can bias estimates. By allowing a distribution of microstructural values to be assigned to a voxel, that is, the number of values is equal to the number of unique streamlines passing through the voxel and retained for analysis, COMMIT offers a more quantitative estimation of network properties. In the context of graph theory, we can capture the dynamic strengthening and weakening of connections based on their underlying microstructure, known to mature rapidly through childhood and adolescence.

Indeed, when repeating the analysis of age-related differences in network properties using the reconstructed number of streamlines (NOS) as edge weights, we observed differences in results. Age-related increases in network properties were present in the fronto-parietal and somatomotor networks but absent from the visual and default mode networks (Supporting Information Table S7). Upon further investigation, we observed a significant positive relationship between age and the number of reconstructed streamlines in the fronto-parietal and somatomotor networks (Supporting Information Table S8)—suggesting that the total NOS may be driving these age-related increases in network properties. Various factors unrelated to the underlying microstructure, such as tract shape, length, and curvature, can impact the number of streamlines reconstructed (Jones et al., 2013; Maier-Hein et al., 2017). One example is depicted in Figure 2C; in the visual network the Meyer’s loop of the optic radiation contains fibers that undergo large turns, which can result in a smaller number of valid streamlines recovered by tractography and many false positives (Chamberland et al., 2018). As such, we need to exercise caution when interpreting results using connectomes weighted by NOS.

Overall, the COMMIT framework offers a nuanced and detailed characterization of microstructural properties along individual streamlines, countering complex intra-voxel heterogeneity, making it a powerful tool for a more meaningful assessment of brain connectivity (Gabusi et al., 2022; Schiavi et al., 2022; Schiavi, Ocampo-Pineda, et al., 2020; Schiavi, Petracca, et al., 2020).

### Limitations and Future Directions

It is important to acknowledge that certain functional networks utilized in our study here contain fewer nodes than others, potentially influencing our interpretations. Although we adopted a robust method to generate reproducible cortical nodes for each functional network, it resulted in some networks having a small number of nodes. Using a parcellation method with finer granularity (Glasser et al., 2016; Schaefer et al., 2017) and replicating analyses in a larger independent cohort such as the adolescent brain development cohort (Casey et al., 2018) would be warranted.

While there is a certain relationship between brain structure and function, structure-function coupling occurs in a spatially dependent hierarchical manner (Baum et al., 2020). The brain is a complex and dynamic organ, with function influenced by a variety of factors, including structural organization (Chamberland et al., 2017) and neural activity. Combining task-based or resting-state fMRI with microstructure-informed connectomes may better elucidate structure-function coupling across the developing brain (Suárez et al., 2020).

Despite running a “gold-standard” dMRI preprocessing pipeline, susceptibility-induced distortion artifacts may introduce an additional source of variance into the diffusion MRI data, especially in fronto-parietal regions with an air/bone interface such as the nasal cavity. While the aforementioned factors may help explain why we did not observe an age dependence of network-based measures of brain connectivity in regions known to remodel in adolescence (e.g., the fronto-parietal network), it is known that functional networks that are in close range demonstrate stronger white matter connectivity (Hermundstad et al., 2013), which may explain why our findings of global efficiency and mean strength were confined to the somatomotor and visual networks. Future work could involve examining changes in edge weight and connection density of short versus long-range connections in younger versus older participants, which might reveal other interesting changes in topology.

Finally, new frontiers in characterizing the developing connectome using biologically meaningful mathematical models of brain connections are promising (Akarca et al., 2023; Seguin et al., 2023). Recent updates to the COMMIT framework offer the opportunity to incorporate additional imaging contrasts, such as myelin-sensitive contrasts, leading to improved delineation of anatomically accurate whole-brain tractography (Leppert et al., 2023; Schiavi et al., 2022).

## CONCLUSION

Incorporating microstructural information into network analyses has shed light on distinct regional age-related development of brain networks. Notably, we observed unique characteristics within the visual network throughout development, supporting its ongoing maturation, reaffirming previously reported patterns of protracted development in the dorsal and ventral visual pathways. Overall, our study demonstrates the power of microstructure-informed tractography to decipher intricate developmental patterns, reinforcing the potential for deepening our understanding of brain connectivity and development.

## ACKNOWLEDGMENTS

We are grateful to the participants and their families for their participation in this study. We thank Umesh Rudrapatna and John Evans for their support with image acquisition protocols, Isobel Ward for assistance with data collection, Joseph Yang for scientific discussions, and Greg Parker for contributions to data preprocessing and model fitting pipelines. Image credit (Figure 1A) by kjpargeter on Freepik. The data were acquired at the UK National Facility for In Vivo MR Imaging of Human Tissue Microstructure funded by the EPSRC (Grant No. EP/M029778/1), and the Wolfson Foundation. SG acknowledges the support of the Royal Children’s Hospital, Murdoch Children’s Research Institute, the University of Melbourne Department of Paediatrics, and the Victorian government’s Operational Infrastructure Support Program.

## SUPPORTING INFORMATION

Supporting information for this article is available at https://doi.org/10.1162/netn_a_00378.

## AUTHOR CONTRIBUTIONS

Sila Genc: Conceptualization; Data curation; Formal analysis; Visualization; Writing – original draft; Writing – review & editing. Simona Schiavi: Conceptualization; Formal analysis; Visualization; Writing – original draft; Writing – review & editing. Maxime Chamberland: Formal analysis; Writing – review & editing. Chantal Tax: Formal analysis; Writing – review & editing. Erika Raven: Data curation; Writing – review & editing. Alessandro Daducci: Funding acquisition; Writing – review & editing. Derek Jones: Conceptualization; Funding acquisition; Writing – original draft; Writing – review & editing.

## FUNDING INFORMATION

Derek Jones, Wellcome Trust (https://dx.doi.org/10.13039/100010269), Award ID: 096646/Z/11/Z. Derek Jones, Wellcome Trust (https://dx.doi.org/10.13039/100010269), Award ID: 104943/Z/14/Z. Chantal Tax, Wellcome Trust (https://dx.doi.org/10.13039/100010269), Award ID: 215944/Z/19/Z. Derek Jones, Engineering and Physical Sciences Research Council (https://dx.doi.org/10.13039/501100000266), Award ID: EP/M029778/1. Chantal Tax, Nederlandse Organisatie voor Wetenschappelijk Onderzoek (https://dx.doi.org/10.13039/501100003246), Award ID: 17331.

## CODE AVAILABILITY

The code for COMMIT is open source and freely available at https://github.com/daducci/COMMIT.

## Supplementary Material



## TECHNICAL TERMS

Diffusion magnetic resonance imaging (dMRI): An MRI acquisition technique sensitised to the movement of water.
Structural connectivity: The physical connectedness of brain structures, via anatomical pathways such as white matter fibers.
Microstructure-informed tractography: A method that incorporates diffusion-weighted imaging data to weight brain networks and obtain a more biologically relevant assessment of brain connectivity.
Intra-axonal signal fraction: The proportion of diffusion-weighted signal arising from inside axons.
Cortical parcellation: A division of brain structure corresponding to anatomical landmarks.
Canonical cortical networks: Interconnected brain networks of known functional relevance.
