# Supplementary material for: Developmental differences in canonical cortical networks: Insights from microstructure-informed tractography
Source: Netw Neurosci. 2024 Oct 1;8(3):946–64. doi: 10.1162/netn_a_00378 (PMC11424039; doi:10.1162/netn_a_00378)
Supplement: Supplementary file 1 [file netn-8-3-946-s001.pdf]

9. Supplementary

9.1. Information

A total of 88 children (Mean age = 12.6, SD = 2.9 years, range= 8 – 19 years) were included in the current study (46 female). Figure S1 shows the age distribution of the cohort. The attending parent was asked to complete a brief survey on their demographics and educational attainment. Majority of parents (69/88) had completed a university degree (78%), 11 completed a certificate or diploma (13%) and 8 respondents completed year 12 or less (9%). The Strengths and Difficulties Questionnaire (SDQ) was administered as a measure of emotional/behavioural difficulties (Goodman, 1997). In a subsample of children and adolescents (N=79, 40 males, 39 females), parent-reported total scores (summation of all SDQ modules) were generally low (mean=6.45, SD=3.90, range=0-19) suggesting low levels of internalising and externalising problems in the cohort.

9.2. Figures

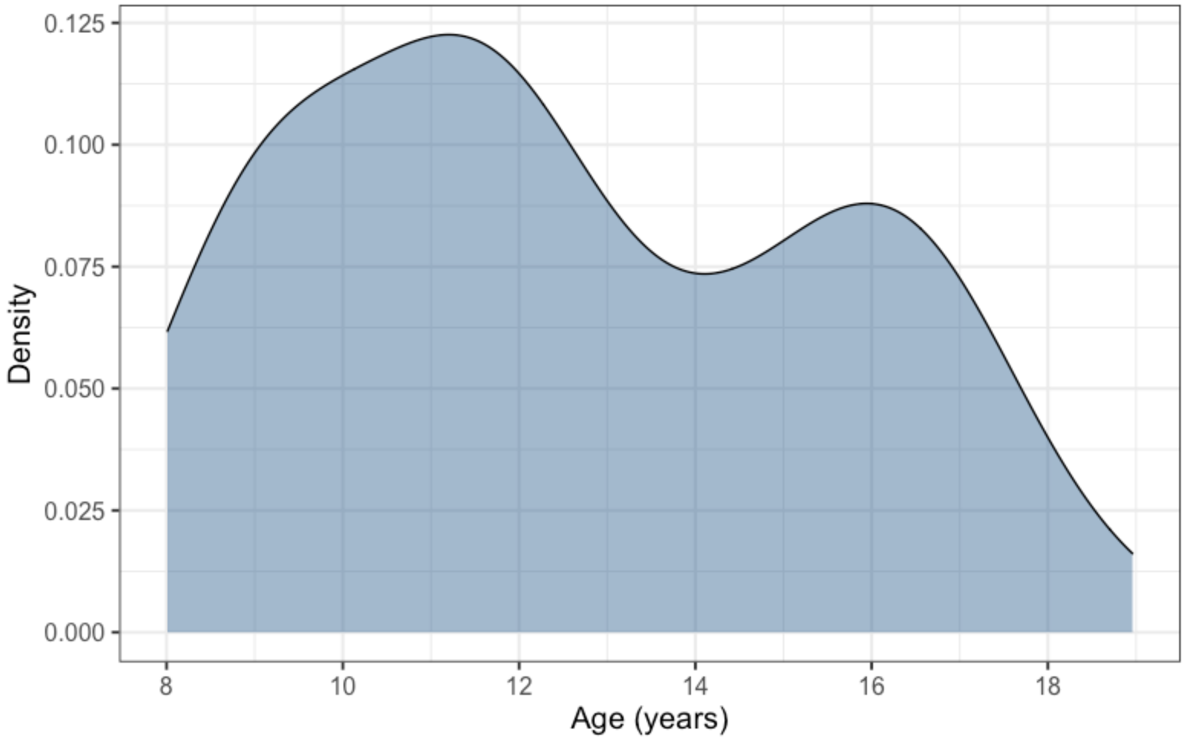

Figure S1: Age distribution of cohort.

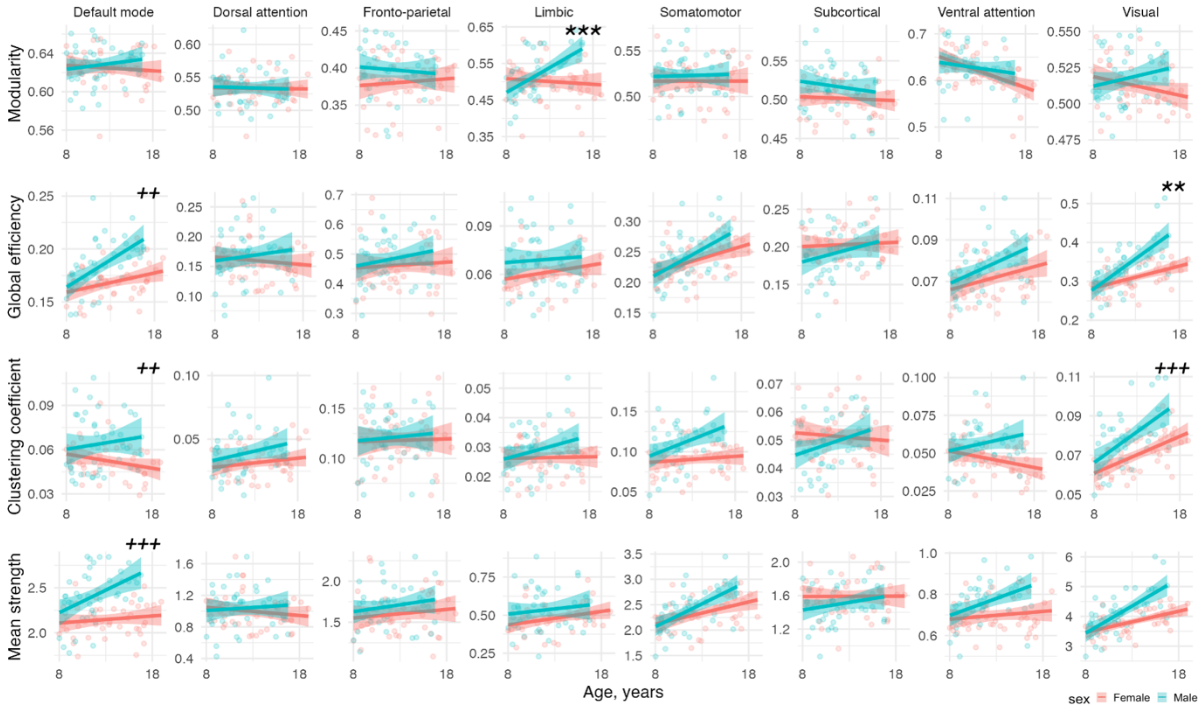

Figure S2: Sex differences in network properties over age. Associations with network measures are annotated in terms of difference in absolute values (main effect: +++=p<.005, ++=p<.01) and in slope over age (interaction term: ***=p<.005, **=p<.01). Red: female, blue: male.

9.3. Tables

Table S1: Voxel-wise diffusivity parameters estimated in a white matter mask for one younger (8-year-old) and one older (17-year-old) participant. Values are reported as mean (SD).

	d_a	d_{par}	d_{perp}
Younger	2.27 (0.71)	2.01 (0.57)	0.61 (0.28)
Older	2.35 (0.62)	1.71 (0.58)	0.62 (0.27)

Table S2: Regions from the Destrieux parcellation assigned to each canonical cortical network. Results for left hemisphere shown (equivalent in right hemisphere). Only nodes overlapping the same network in >80% of participants were included in the analysis.

Region	Name	X	Y	Z	Yeo7_name
2	G_and_S_occipital_inf	23	60	180	visual
3	G_and_S_paracentral	63	100	60	somatomotor
4	G_and_S_subcentral	63	20	220	somatomotor
5	G_and_S_transv_frontopol	13	0	250	dmn
6	G_and_S_cingul-Ant	26	60	0	dmn
7	G_and_S_cingul-Mid-Ant	26	60	75	ventralattention
9	G_cingul-Post-dorsal	25	60	250	dmn
10	G_cingul-Post-ventral	60	25	25	dmn
11	G_cuneus	180	20	20	visual
12	G_front_inf-Opercular	220	20	100	ventralattention
13	G_front_inf-Orbital	140	60	60	dmn
15	G_front_middle	140	100	180	frontoparietal
16	G_front_sup	180	20	140	dmn
17	G_Ins_lg_and_S_cent_ins	23	10	10	ventralattention
18	G_insular_short	225	140	140	ventralattention
19	G_occipital_middle	180	60	180	visual
20	G_occipital_sup	20	220	60	visual
21	G_oc-temp_lat-fusifor	60	20	140	visual
22	G_oc-temp_med-Lingual	220	180	140	visual
23	G_oc-temp_med-Parahip	65	100	20	limbic
24	G_orbital	220	60	20	limbic
25	G_pariet_inf-Angular	20	60	220	dmn
26	G_pariet_inf-Supramar	100	100	60	ventralattention
27	G_parietal_sup	220	180	220	dorsalattention
28	G_postcentral	20	180	140	somatomotor
29	G_precentral	60	140	180	somatomotor
31	G_rectus	20	60	100	limbic
32	G_subcallosal	60	220	20	limbic
33	G_temp_sup-G_T_transv	60	60	220	somatomotor
35	G_temp_sup-Plan_polar	65	220	60	limbic
38	G_temporal_middle	180	60	60	dmn
41	Lat_Fis-post	61	60	100	somatomotor
42	Pole_occipital	140	20	60	visual
43	Pole_temporal	220	180	20	limbic
44	S_calcarine	63	180	180	visual
45	S_central	221	20	10	somatomotor
46	S_cingul-Marginalis	221	20	100	ventralattention
48	S_circular_insula_inf	221	20	220	ventralattention
49	S_circular_insula_sup	61	220	220	ventralattention
50	S_collat_transv_ant	100	200	200	limbic
51	S_collat_transv_post	10	200	200	visual
52	S_front_inf	221	220	20	frontoparietal

56	S_intrapariet_and_P_trans	143	20	220	dorsalattention
57	S_oc_middle_and_Lunatus	101	60	220	visual
58	S_oc_sup_and_transversal	21	20	140	visual
60	S_oc-temp_lat	221	140	20	dorsalattention
61	S_oc-temp_med_and_Lingual	141	100	220	visual
62	S_orbital_lateral	221	100	20	frontoparietal
63	S_orbital_med-olfact	181	200	20	limbic
65	S_parieto_occipital	101	100	180	visual
69	S_precentral-sup-part	21	20	200	dorsalattention
71	S_subparietal	101	60	60	dmn
73	S_temporal_sup	223	220	60	dmn
74	S_temporal_transverse	221	60	60	somatomotor
76	Left-Thalamus-Proper	0	118	14	subcortical
77	Left-Caudate	122	186	220	subcortical
78	Left-Putamen	236	13	176	subcortical
79	Left-Pallidum	12	48	255	subcortical
80	Left-Hippocampus	220	216	20	subcortical
81	Left-Amygdala	103	255	255	subcortical
82	Left-Accumbens-area	255	165	0	subcortical

Table S3: Results of mixed-effect model selection for first level global graph network analysis. Values reported are Akaike Information Criterion (AIC) of each model fit.

Model	Modularity	Global Efficiency	Clustering Coefficient	Mean Strength
M1a	-2816.78	-2549.70	-3792.68	244.49
M2a	-2821.03	-2553.69	-3796.30	237.85
M3a	-2832.02	-2565.74	-3795.91	167.79
M4a	-2860.55	-2575.29	-3814.42	90.46
M1b	-2825.11	-2569.99	-3801.41	215.66
M2b	-2826.42	-2570.17	-3802.19	214.00
M3b	-2840.35	-2586.03	-3804.65	138.97
M4b	**-2865.94***	**-2591.77***	**-3820.31***	**66.60***

Note: Bold indicates lowest AIC for each graph measure; * indicates if the age by network term was significant at p<.005

Footnote: Models tested are as follows:
M1a <- lmer(measure ~ age + sex + network + (1|ID), REML=FALSE, data=data)
M2a <- lmer(measure ~ age * sex + network + (1|ID), REML=FALSE, data=data)
M3a <- lmer(measure ~ age * network + sex + (1|ID), REML=FALSE, data=data)
M4a <- lmer(measure ~ age * sex * network + (1|ID), REML=FALSE, data=data)
M1b <- lmer(measure ~ age + sex + network + ICV + (1|ID), REML=FALSE, data=data)
M2b <- lmer(measure ~ age * sex + network + ICV + (1|ID), REML=FALSE, data=data)
M3b <- lmer(measure ~ age * network + sex + ICV + (1|ID), REML=FALSE, data=data)
M4b <- lmer(measure ~ age * sex * network + ICV + (1|ID), REML=FALSE, data=data)

Table S4: Summary statistics for the relationship between age and global sub-network characteristics, adjusted for connection density.

**Network**	**Modularity**		**Global efficiency**		**Clustering coefficient**		**Mean strength**	
	R^2	**p-value**	R^2	**p-value**	R^2	**p-value**	R^2	**p-value**
Default mode	0.07	0.91	0.37	**0.003**	0.63	0.06	0.43	0.10
Dorsal attention	-0.05	0.82	0.06	0.41	0.16	0.19	0.05	0.24
Fronto-parietal	0.13	0.68	0.12	0.59	0.00	0.95	0.17	0.52
Limbic	0.08	0.09	0.24	0.80	0.20	0.53	0.31	0.87
Somatomotor	0.08	0.70	0.27	**0.001**	0.63	**< .001**	0.33	**< .001**
Subcortical	0.20	0.21	0.03	0.25	0.11	0.74	0.01	0.47
Ventral attention	0.14	**0.002**	0.19	0.02	0.42	0.22	0.23	0.07
Visual	0.19	0.05	0.43	**< .001**	0.57	**< .001**	0.46	**< .001**

Note: Adjusted R^2 determined using a linear model including age, sex, total intracranial volume and connection density. Bold values indicate p<.005.

Table S5: Results from comparison of age-associations of graph measures with reference to the visual network. Bold values indicate networks which have significantly different slopes to the age-relationship in the visual network, generated using linear mixed efforts models.

**Network**	**Global efficiency**		**Clustering coefficient**		**Mean strength**	
	**t**	**p-value**	**t**	**p-value**	**t**	**p-value**
Visual (reference)						
Default mode	-1.65	.10	-2.91	**.004**	-4.08	**< .001**
Dorsal attention	-2.96	**.003**	-1.17	.24	-5.25	**< .001**
Fronto-parietal	-1.64	.10	-1.66	.10	-3.91	**< .001**
Limbic	-2.04	.04	-1.87	.06	-4.01	**< .001**
Somatomotor	-0.60	.55	-1.16	.25	-1.78	.08
Subcortical	-2.19	.03	-2.19	.03	-4.55	**< .001**
Ventral attention	-1.93	.05	-3.05	**.002**	-4.34	**< .001**
Somatomotor (reference)						
Default mode	-1.05	.29	-1.05	.29	-2.30	.02
Dorsal attention	-2.36	.02	-2.36	.02	-3.48	**< .001**
Fronto-parietal	-1.04	.30	-1.04	.30	-2.13	.03
Limbic	-1.45	.15	-1.45	.15	-2.23	.03
Subcortical	-1.60	.11	-1.60	.11	-2.78	.006
Ventral attention	-1.33	.18	-1.33	.18	-2.56	.011
Visual	0.60	.55	0.60	.55	1.78	.08

Note: model used was the best fitting model deduced from Table S3.

Table S6: Summary statistics for the relationship between age and network statistics computed in parcels obtained from the Desikan Killany atlas for five distinct lobes.

Lobe	Modularity		Global efficiency		Clustering coefficient		Mean strength	
	R^2	p-value	R^2	p-value	R^2	p-value	R^2	p-value
Frontal	0.12	0.82	0.53	**< .001**†	0.52	**< .001**	0.60	**< .001**†
Parietal	0.09	0.02	0.42	**0.002**	0.38	**0.001**	0.45	**< .001**†
Temporal	0.06	0.07	0.33	0.25	0.52	0.90	0.36	0.19
Occipital	0.05	0.21	0.25	**0.002**†	0.15	0.03	0.29	**0.001**†
Subcortical	0.21	0.22	0.03	0.24	0.10	0.76	0.01	0.47

Note: Adjusted R^2 determined using a linear model including age, sex, total intracranial volume and connection density. Bold values indicate p<.005. † indicates statistically significant results without connection density as a covariate in the linear model.

Table S7: Summary statistics for the relationship between age and global sub-network characteristics, adjusted for connection density. Computed using number of streamlines without COMMIT.

Network	Modularity		Global efficiency		Clustering coefficient		Mean strength	
	R^2	p-value	R^2	p-value	R^2	p-value	R^2	p-value
Default mode	.13	.12	-.01	.32	.23	.50	-.03	.42
Dorsal attention	.08	.05	.08	.49	.03	.57	.07	.49
Fronto-parietal	.30	**< .001**	.33	**.002**	.38	**.004**	.42	**< .001**
Limbic	-.01	.15	.19	.34	.11	.56	.19	.52
Somatomotor	.11	.32	.25	**< .001**	.08	.02	.27	**.003**
Subcortical	.27	**< .001**	.02	.77	.06	.34	.02	.61
Ventral attention	.28	.07	.17	.85	.23	.26	.02	.68
Visual	.15	.95	.00	.93	.01	.94	.02	.98

Table S8: Summary statistics for the relationship between age and number of reconstructed streamlines without COMMIT.

Network	Number of streamlines	
	R^2	p-value
Raw whole-brain	**0.22**	**< .001**
Default mode	-0.01	0.49
Dorsal attention	0.01	0.23
Fronto-parietal	**0.19**	**< .001**
Limbic	0.06	0.02
Somatomotor	**0.23**	**< .001**
Subcortical	0.03	0.07
Ventral attention	-0.01	0.50
Visual	-0.01	0.72
